# Assessment of the relationship between central venous pressure waveform and the severity of tricuspid valve regurgitation using data science

**DOI:** 10.1038/s41598-024-74890-8

**Published:** 2024-10-22

**Authors:** Shinichi Akabane, Masaaki Asamoto, Seiichi Azuma, Mikiya Otsuji, Kanji Uchida

**Affiliations:** 1 Department of Anesthesiology and Pain Relief Center, The University of Tokyo Hospital, Tokyo, Japan; 2 Department of Anesthesiology, Tokyo Teishin Hospital, Tokyo, Japan

**Keywords:** Valvular disease, Valvular disease, Blood flow

## Abstract

Purpose The relationship between the height of the V wave in the central venous pressure (CVP) waveform and the severity of tricuspid regurgitation (TR) is well known. Their diagnostic ability is unconfirmed. This study explored CVP waveform variations with TR. Methods All patients who underwent preoperative echocardiography and CVP waveform measurements before surgery at our institution were included. Indices were created to capture each feature of the CVP waveform. The median value for each case was obtained and statistically analyzed according to the severity of TR. A deep learning technique, Transformer, was used to handle the complex features of CVP waveforms. Results This study included 436 cases. The values for C wave – Y descent, X descent – Y descent, and V wave – Y descent differed significantly in the Jonckheere–Terpstra test (p = 0.0018, 0.027, and 0.077, respectively). The area under the receiver operating characteristic (ROC) curve (AUC) for X descent – Y descent in two groups, none to moderate TR and severe TR, was 0.83 (95% confidence interval (CI) [0.68, 0.98]). For Transformer, the accuracy of the validation dataset was 0.97. Conclusions The shape of the CVP waveform varied with the severity of TR in a large dataset.

**Supplementary Information:**

The online version contains supplementary material available at 10.1038/s41598-024-74890-8.

## Introduction

Central venous catheter placement and central venous pressure (CVP) measurements are commonly performed in operating rooms and intensive care units^1^. Several reports have been published on CVP values and their diagnostic benefits in clinical situations^2,3^. CVP and CVP waveform diagnoses are routinely performed in clinical practice. There are reports that intraoperative severe tricuspid regurgitation (TR) is associated with long-term mortality after cardiac surgery^4,5^. It is known that the waveform of CVP changes according to the severity of TR. Specifically, the large V wave of the CVP waveform that occurs during TR is well known. A large V wave is observed owing to the holosystolic nature of regurgitation. Moreover, the C wave is higher than the A wave, and the V wave merges with the C wave, resulting in an elevated X descent. However, the incidence of a large V wave has been reported in a few reports; these were case reports, and their findings were empirical. As a review of the literature did not yield any large-scale studies on the diagnostic ability of CVP waveform^4–17^, we considered it necessary to verify this.

Related work is limited. There are some case reports about the relationship between CVP waveform and TR^9–17^. These case reports describe giant C-V waves associated with TR. However, each report only includes one case. There are some reviews about the relationship between CVP waveform and TR^6–8^. These review mentions TR and CVP waveforms, but it must be said that these descriptions are largely based on empirical knowledge and lack supporting papers or data. Our literature search indicates that there have been no statistical analyses or deep learning analyses on TR and CVP waveforms to date. There are few studies describing the relationship between CVP waveform and TR, but some research has investigated the severity of intraoperative TR and long-term mortality in cardiac surgery^4,5^. It seems meaningful to estimate the severity of TR intraoperatively.

This study investigated whether the shape of the CVP waveform differed according to the severity of TR. The purpose of this study was to verify the relationship between CVP waveform and TR severity, which had only been known empirically, and to identify features that can be distinguished. This study is divided into two phases. In the first phase, we used statistical analysis to verify whether the previously discussed relationship between CVP waveform and TR severity is correct. In the second phase, we decided to use deep learning to uncover previously unidentified relationships. Among the numerous deep learning methods, we adopted one that can handle time series data and visualize the basis of its decisions. We considered the Transformer as the deep learning method and the Attention map for visualizing its decision-making process to be suitable for this study.

Simple indices were created from the CVP waveform, and statistical analysis was performed according to the severity of TR. Although the simple indices were based on the hypotheses mentioned above, these indices do not always adequately reflect the severity of TR.

Three simple indices that reflect large V waves, X descent, and C waves were created from the CVP waveforms in the present study. The Y descent is not affected by TR; therefore, the height of the V wave from the Y descent, the height of the X descent from the Y descent, and the height of the C wave from the Y descent were analyzed in this study to determine whether these indices of pressure differences were statistically significant according to the severity of TR.

Deep learning was also used in this study as it enables the inclusion of complex features. Deep learning is an effective method when the appropriate features reflecting the severity of TR are not known in advance. Explainable AI (XAI) was used in this study to visualize the deep learning decision basis as it can reveal parts of the CVP waveform that are related to the severity of TR. For this study, we adopted the Transformer as the deep learning method because it can handle time series data and supports XAI.

A deep learning method, Transformer in Time Series, was used in this study^18^. CVP waveforms were normalized to the maximum and minimum values and then analyzed using Transformer in Time Series. Rather than analyzing the absolute pressure differences, Transformer in Time Series analyzes the relative shapes of the waveforms. The shape of the CVP waveform would be of interest if a suitable model can be developed using deep learning. XAI was used to visualize the important parts of the shape, and an attention map was used to visualize the decision base of the Transformer in Time Series^19^.

In the past, Recurrent Neural Network (RNN) and Long Short Term Memory (LSTM) models have demonstrated high accuracy in predicting clinical events, but they were not easily interpretable^20–22^. Subsequently, efforts were made to enhance interpretability by incorporating attention mechanisms into RNN and LSTM models^23,24^. More recently, attempts have been made to improve interpretability using Transformer models^25^. Since the attention mechanism is inherently integrated into the Transformer architecture and requires no special implementation, we considered it reasonable to use Transformers in our approach.

This study investigated whether the shape of the CVP waveform differed according to the severity of TR. The goal is to use statistics to confirm previously known insights and to investigate previously unnoticed features using deep learning, in order to extract features that can be managed by humans.

## Materials and methods

### Ethics

This observational study was approved by the Research Ethics Committee of the Faculty of Medicine at the University of Tokyo (ID: 2023166NI). All methods were performed in accordance with the Declaration of Helsinki, particularly adhering to item 32 which states: ‘For medical research using identifiable human material or data, such as research on material or data contained in biobanks or similar repositories, physicians must seek informed consent for its collection, storage and/or reuse. There may be exceptional situations where consent would be impossible or impracticable to obtain for such research. In such situations, the research may be done only after consideration and approval of a research ethics committee.’ We provided opt-out methods for the participants by publishing a summary of this study on our university website^26,27^. Informed consent was waived by the Research Ethics Committee of the Faculty of Medicine at the University of Tokyo in accordance with the Ethical Guidelines for Medical and Health Research Involving Human Subjects in Japan. This manuscript was written in accordance with DECIDE-AI^28^.

### Data collection

Among the patients who underwent surgery under general anesthesia at the University of Tokyo Hospital between April 2021 and June 2023, those who underwent preoperative echocardiography, intraoperative CV catheter insertion, and pressure waveform measurements before the start of surgery were included in this study. The waveforms displayed on the biometric monitor (CSM-1000 series, Nihon Kohden) intraoperatively were analyzed backward using the database of the recorded cases. The waveform sampling rate was 250 Hz. Patients with atrial fibrillation (AF) or atrial flutter (AFL) in the echocardiogram (ECG) rhythm were excluded owing to the absence of A waves. The CVP waveform before the start of surgery was used to eliminate any influence of the surgery.

### Programming

The programs used for the analysis in this study were created using Python (version 3.9.12) and R (version 4.1.3). The Python libraries used in this study included NumPy (version 1.21.6), SciPy (version 1.8.0), pandas (version 1.4.2), matplotlib (version 3.5.2), seaborn (version 0.11.2), scikit-learn (version 1.1.0), tslearn (version 0.5.3.2), TensorFlow (version 2.9.1), and Keras (version 2.9.0). The R library used was PMCMRplus (version 1.9.6).

### Data cleaning by k-Shape

We extracted beat-by-beat CVP waveforms corresponding to the interval from the $R\;wave\;time-\;RR\;time\;\times5/16$ to the $\:next\:R\:wave\:time\:-\:RR\:time\times\:1/16$ of the ECG as one beat such that the information from one P wave to the subsequent P wave was included with a margin.

The removal of CVP waveform artifacts^29^ was performed using k-Shape, which removed the CVP waveform artifacts via anomaly detection^30,31^, as described in Appendix 1.

Diagrams were created to evaluate the waveforms after the removal of the artifacts. All CVP waveforms were included after the removal of artifacts. The CVP waveform underwent resizing to standardize the time scale and normalize the maximum and minimum pressure values.

**Fig. 1:**
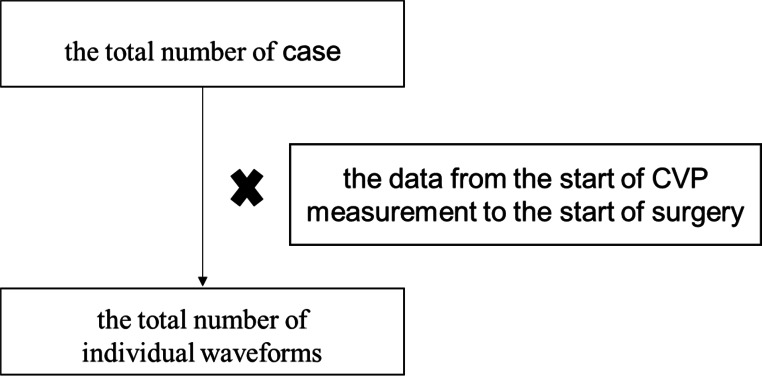
We are collecting CVP waveforms for each case from the start of CVP measurement to the beginning of the surgery.

We are using the data from the start of CVP measurement to the start of surgery for each case. Therefore, the total number of waveforms is greater than the number of cases (Fig. 1).

### Detection of CVP waveform vertices

The local maximum and local minimum of the CVP waveform were detected. C wave was defined as the closest local maximum after the R wave of ECG. V wave was defined as the closest local maximum after the apex of the T wave on ECG. X descent was defined as the global minimum between the R wave of ECG and the V wave of CVP. Y descent was defined as the global minimum after the V wave of the CVP.

### Statistical analysis

Statistical analyses were performed to verify each of the CVP waveform indices. Three indices were created and calculated per beat in this study: $\:V\:wave-Y\:descent$, $\:X\:descent-Y\:descent$, and $\:C\:wave-Y\:descent$. The median value within the case was obtained for each index, and was considered representative of the case.

The Jonckheere–Terpstra test was performed for representative values of the indices based on the severity of TR. The Jonckheere-Terpstra test is a non-parametric test used to detect trends, such as whether values increase or decrease monotonically, across three or more groups. We performed the Jonckheere–Terpstra test with the alternative hypothesis that the values of the indices rise with increasing TR severity (one-sided test). The Steel–Dwass multiple comparison test was used to perform multiple testing for indices that differed significantly. The Steel–Dwass multiple comparison test is a non-parametric statistical method used for comparing multiple groups. Receiver operating characteristic (ROC) curves were drawn for the indices that showed a significant trend in the Jonckheere–Terpstra test. The area under the ROC curve (AUC) was calculated subsequently, and the cut-off value was determined using the Youden index. The 95% confidence intervals (CIs) for the AUC were calculated using the Hanley method^32^. The significance level was set at *p* < 0.05.

### Deep learning

CVP waveforms were classified according to the severity of TR. All beat-by-beat waveform data without TR and those with severe TR were used for class classification using deep learning. As mentioned above, we are using the data from the start of CVP measurement to the start of surgery for each case. Therefore, the total number of waveforms is greater than the number of cases. (Fig. 1).

The training and validation data comprised 60% and 40% of the cases, respectively. The CVP waveform was normalized for each beat using Min-Max normalization. The maximum and minimum pressure values were converted to 1 and 0, respectively. Each beat was converted into 500 sequences.

**Fig. 2:**
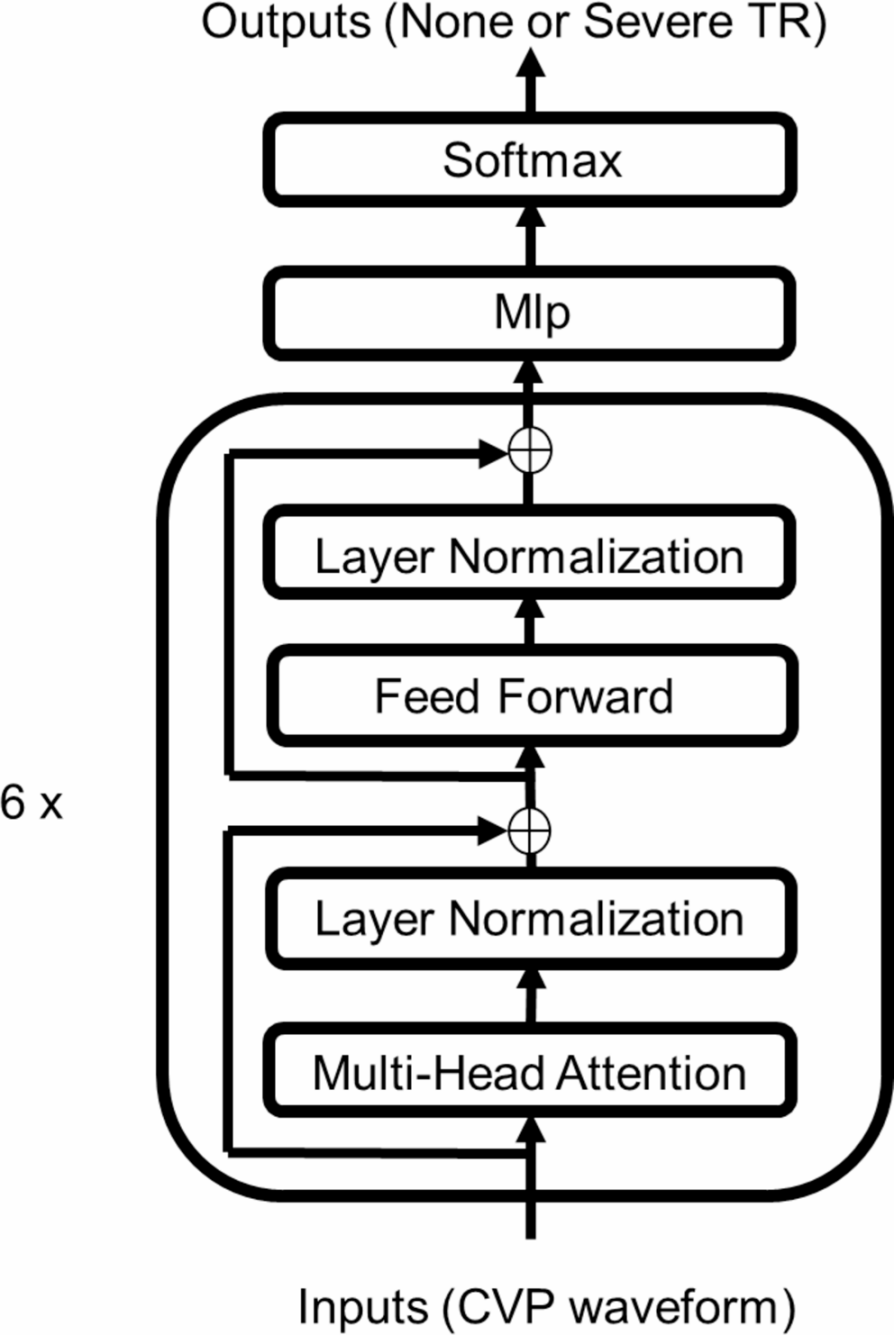
Here is a diagram of the Transformer architecture used in this research. It is composed of a Transformer encoder. Feed Forward, Feed Forward Network; MLP, MultiLayer Perceptron.

The deep learning method Transformer in Time Series^18,33^ was programmed following the example provided in the official Keras documentation^33^. We have illustrated our model architecture with a diagram (Fig. 2). This model consists of a transformer encoder and multilayer perceptron (MLP) units. The transformer encoder consisted of six transformer blocks. The hyperparameters of the multihead attention, which constituted the transformer encoder, were four for the number of attention heads and 32 for the size of each attention head. The Adam optimizer was used. The learning rate, weight decay, batch size, and epoch were 5e-5, 5e-4, 256, and 50, respectively.

The transformer in the Time Series visualized the decision base using an attention map created using attention rollout^19^. Keras documentation was used for programming^34^. We calculated attention in the same way as in the original paper^18^. Attention scores can be obtained by setting return_attention_scores to True in Keras’ keras.layers.MultiHeadAttention function. Attention map was visualized by applying attention rollout to the attention scores.

## Results

**Fig. 3:**
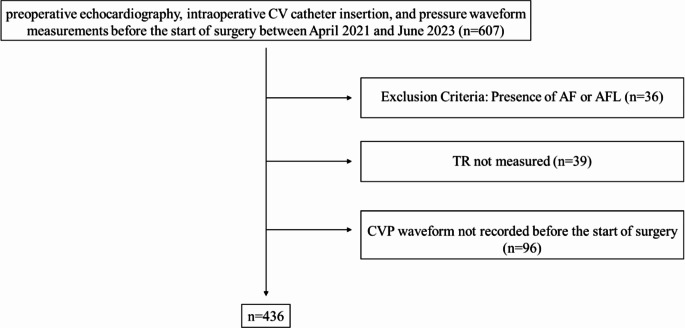
Among the patients who underwent surgery under general anesthesia at the University of Tokyo Hospital between April 2021 and June 2023, those who underwent preoperative echocardiography, intraoperative CV catheter insertion, and pressure waveform measurements were included in this study (*n* = 607). Thirty-six patients had AF or AFL on ECG, and 39 patients underwent echocardiography without TR measurement. Ninety-six patients did not have CVP waveform data before the start of surgery. Consequently, 436 patients were included. CVP, central venous pressure; TR, tricuspid regurgitation; AF, atrial fibrillation; AFL, atrial flutter; ECG, echocardiogram.

Among the total patients who underwent surgery under general anesthesia at the University of Tokyo Hospital between April 2021 and June 2023, 607 underwent preoperative echocardiography and intraoperative CV catheter insertion. Thirty-six patients had AF or AFL on ECG, and 39 patients underwent echocardiography without the measurement of TR. Furthermore, 96 patients did not have a record of their CVP waveform before the start of surgery. Consequently, 436 patients were included in this study (Fig. 3). The median age was 62 years, and the quartile range was [47,78]. This study included 248 males and 188 females. Thirty-one, 271, 99, 24, and 11 patients had absent, trivial, mild, moderate, and severe TR, respectively. A total of 12,292, 106,784, 33,523, 7617, and 8454 beats had absent, trivial, mild, moderate, and severe TR, respectively. The demographic data are presented in Table 1.

**Table 1:** Demographic data.

TR	None	Trivial	Mild	Moderate	Severe	Total
Patients	31 (7%)	271 (62%)	99 (23%)	24 (6%)	11 (3%)	436 (100%)
Male, sex	20 (65%)	162 (60%)	52 (53%)	16 (67%)	6 (55%)	248 (57%)
Female, sex	11 (35%)	109 (40%)	47 (47%)	8 (37%)	5 (45%)	188 (43%)
Age, years	70 [59, 80]	62 [48, 78]	63 [49, 82]	51 [34, 75]	42 [28, 74]	62 [47, 78]
Waves	12,292	106,784	33,523	7617	8454	168,670

Data are presented as median [first quartile, third quartile], or n(%).

**Fig. 4:**
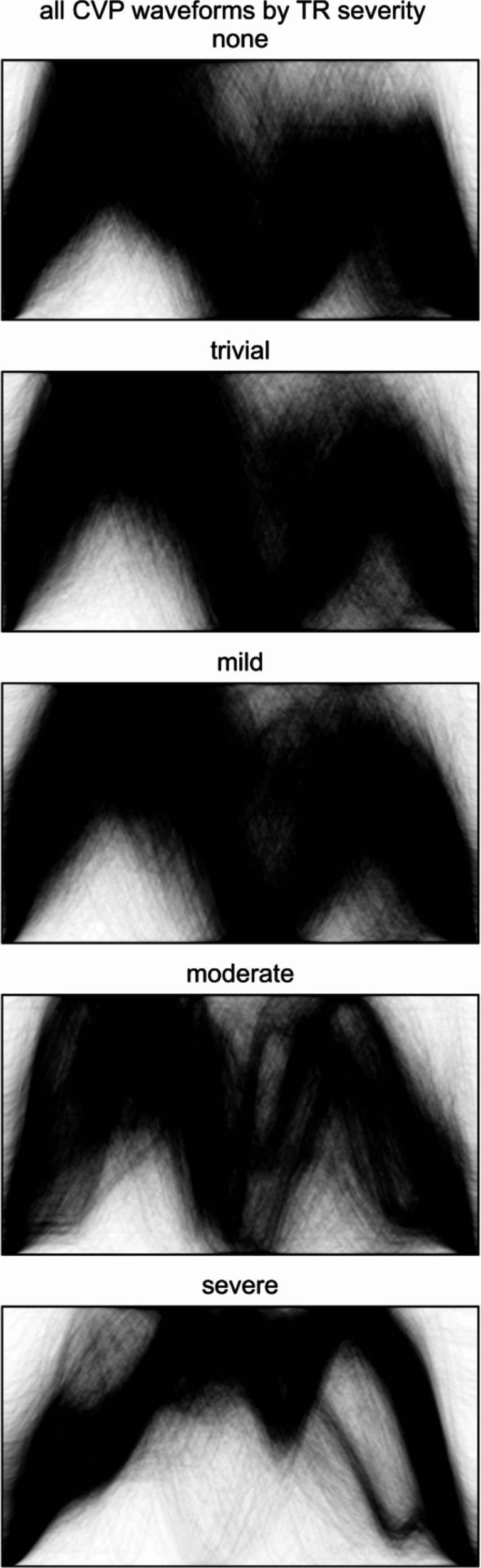
Individual CVP waveforms were drawn in black with increased transparency. All CVP waveforms after the removal of artifacts were included. The CVP waveform underwent resizing to standardize the time scale and normalize the maximum and minimum pressure values. CVP, central venous pressure.

The artifacts were removed from all CVP waveforms using k-Shape. A diagram of the CVP waveforms according to the TR severity after artifact removal is shown in Fig. 4.

**Fig. 5:**
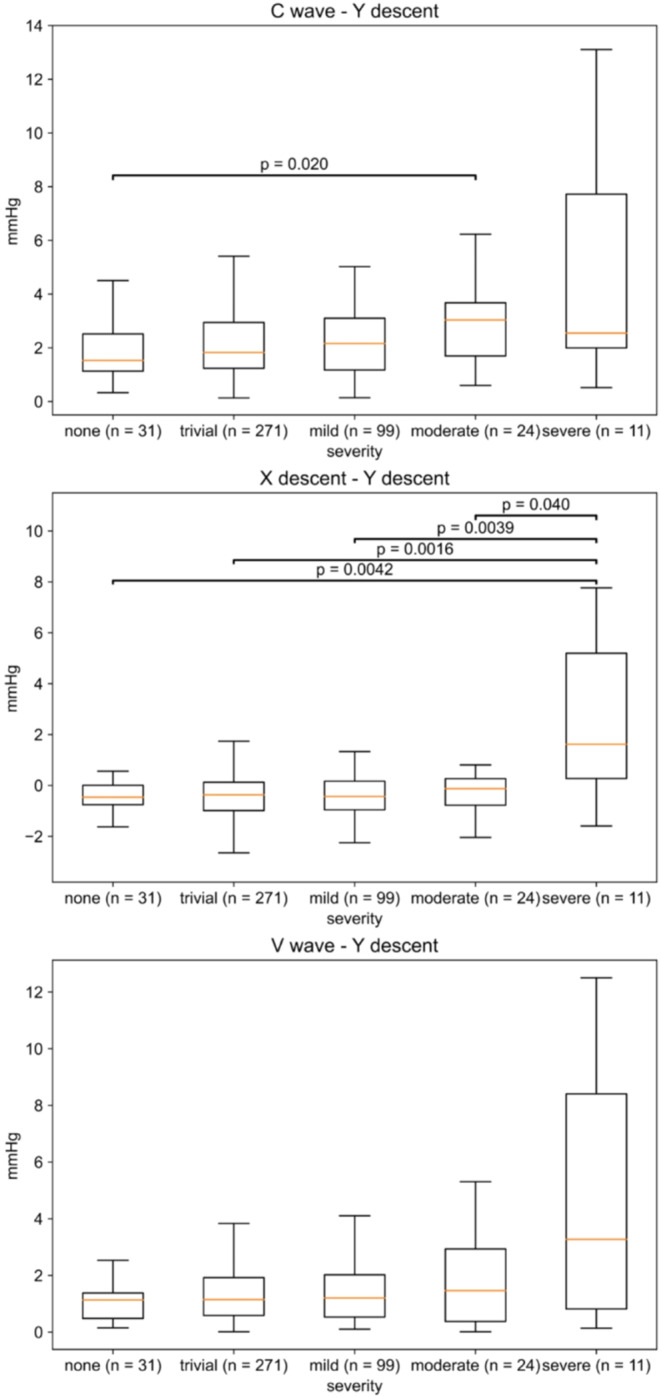
V wave - Y descent, X descent - Y descent, and C wave - Y descent were calculated for each waveform in the cases and the median value was determined for each case. Jonckheere–Terpstra test was performed using these values according to the severity of TR. C wave - Y descent (p value = 0.0018), X descent - Y descent (p value = 0.027), and V wave - Y descent (p value = 0.077). The p-value is written above the bar in cases with a significant difference in Steel–Dwass’s multiple comparison test. TR, tricuspid regurgitation.

$\:C\:wave\:-\:Y\:descent$, $\:X\:descent-Y\:descent$, and $\:V\:wave-Y\:descent$ were statistically analyzed according to the severity of TR to verify each feature of the CVP waveform. Figure 5 presents the results of the analyses. $\:C\:wave\:-\:Y\:descent$ showed a significant trend in the Jonckheere–Terpstra test (*p* = 0.0018). $\:X\:descent-Y\:descent$ also showed a significant trend (*p* = 0.027); however, $\:V\:wave-Y\:descent$ did not show a statistically significant trend (*p* = 0.077). Multiple comparison testing was performed for $\:C\:wave\:-\:Y\:descent$ and $\:X\:descent-Y\:descent$. $\:C\:wave\:-\:Y\:descent$ was found to differ significantly between the none and moderate groups (*p* = 0.020) in the Steel–Dwass’s multiple comparison test. Similarly, $\:X\:descent-Y\:descent$ was found to differ significantly between the none and severe groups (*p* = 0.0042), the trivial and severe groups (*p* = 0.0016), the mild and severe groups (*p* = 0.0039), and the moderate and severe groups (*p* = 0.040) in the Steel–Dwass’s multiple comparison test.

**Fig. 6:**
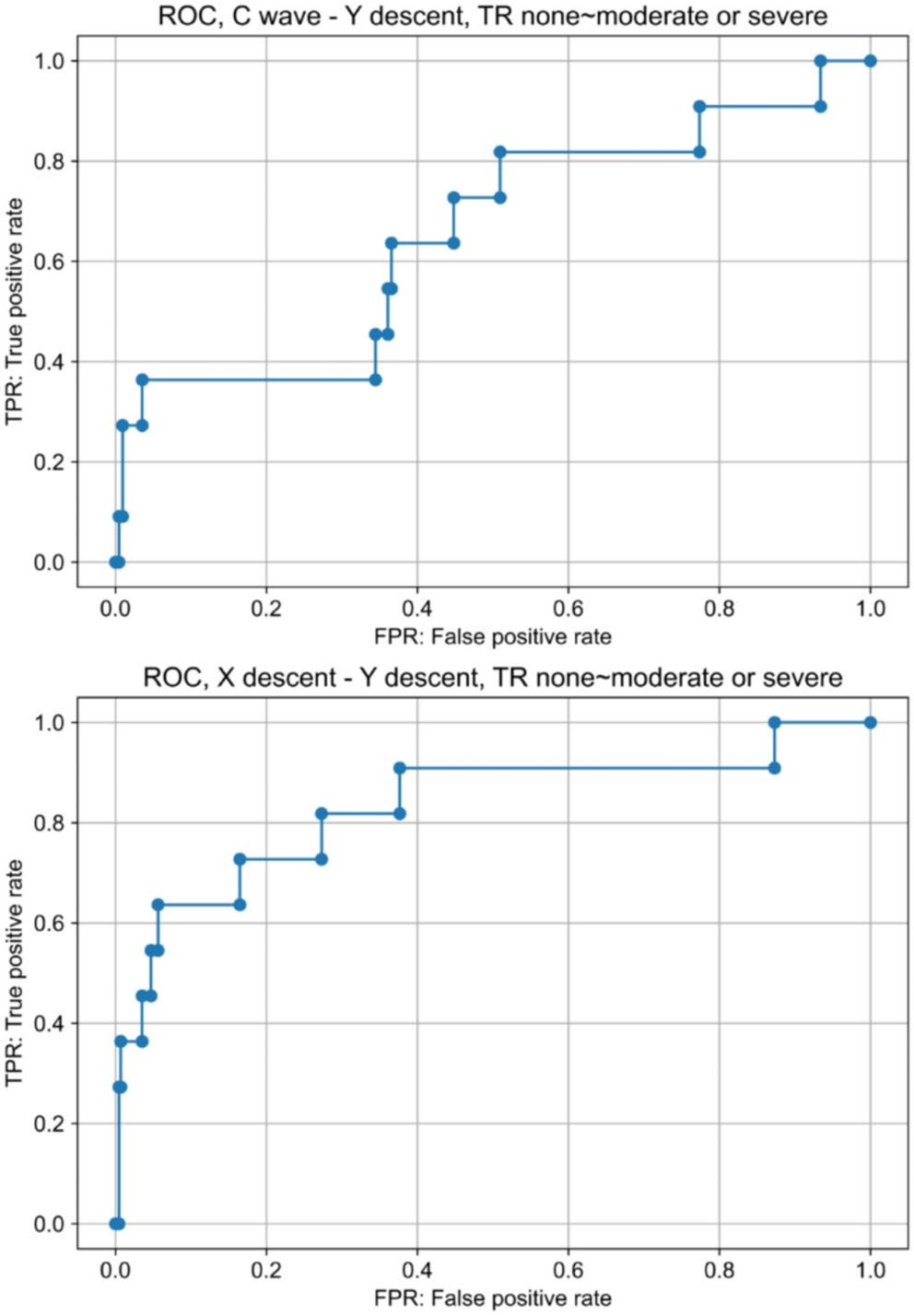
C wave - Y descent and X descent - Y descent were calculated for each waveform in the cases and the median value was determined for each case. Using these values, ROC curve was drawn for the two groups, TR none to moderate and severe. C wave - Y descent (AUC: 0.66, 95% CI by the Hanley method [0.47, 0.83], Youden index: 5.9 mmHg, specificity: 0.96, sensitivity: 0.36). X descent - Y descent (AUC: 0.83, 95% CI by the Hanley method [0.68, 0.98], Youden index: 1.3 mmHg, specificity: 0.94, sensitivity: 0.64). TR, tricuspid regurgitation; AUC, Area under the receiver operating characteristic curve; ROC, receiver operating characteristic curve; CI, confidence interval.

ROC curves were drawn for $\:C\:wave\:-\:Y\:descent$ and $\:X\:descent-Y\:descent$ in the two groups: the TR none to moderate and TR severe groups. The AUC and cut-off values were obtained with the Youden index. The results are presented in Fig. 6. The AUC was 0.65 (95% CI [0.47, 0.83]) for $\:C\:wave-Y\:descent$, and the cut-off value determined by the Youden index was 5.8 mmHg. The specificity and sensitivity were 0.96 and 0.36, respectively. The AUC was 0.83 (95% CI [0.68, 0.98]) for $\:X\:descent-Y\:descent$, and the cut-off value determined by the Youden index was 1.3 mmHg. The specificity and sensitivity were 0.94 and 0.64, respectively.

Deep learning class classification was performed using the TR-none and TR-severe waveform data. The training data included 19 cases with no TR and six cases with severe TR. The validation dataset included 12 cases with no TR and five cases with severe TR. The training data included 9415 beats with no TR and 6219 beats with severe TR. The validation data included 2877 beats with no TR and 2235 with severe TR.

**Fig. 7:**
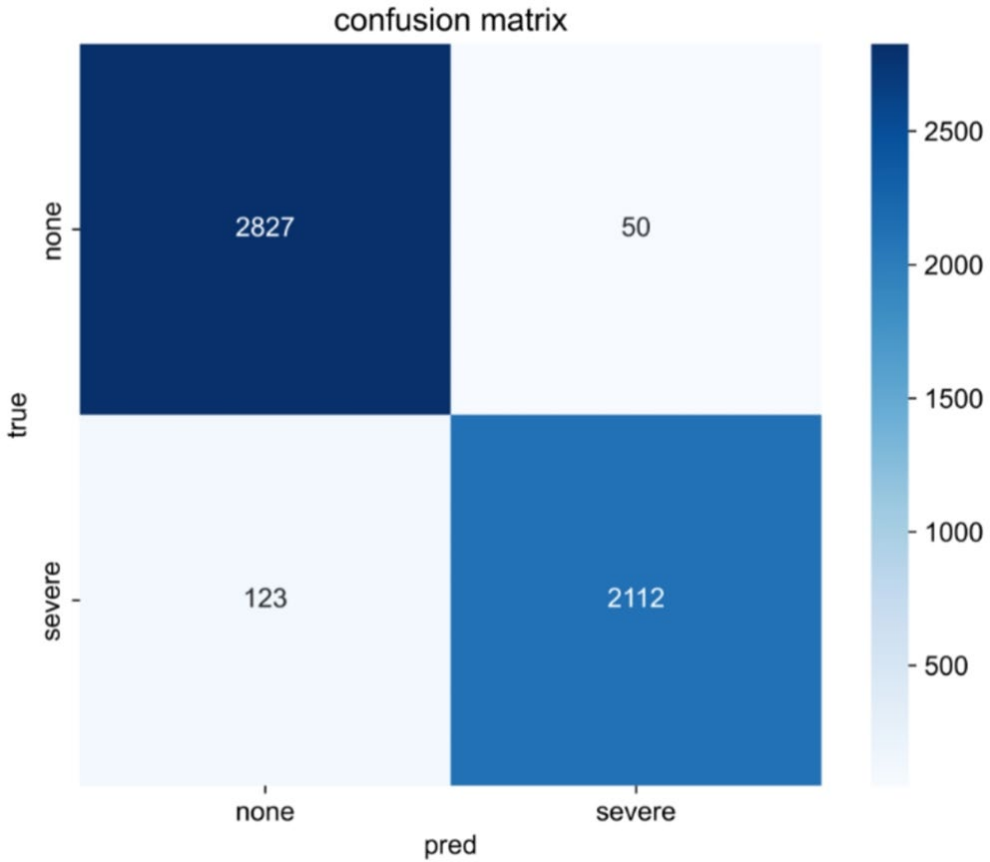
Results of the prediction of the validation data in the model created by Transformer in Time Series with the confusion matrix.

**Fig. 8:**
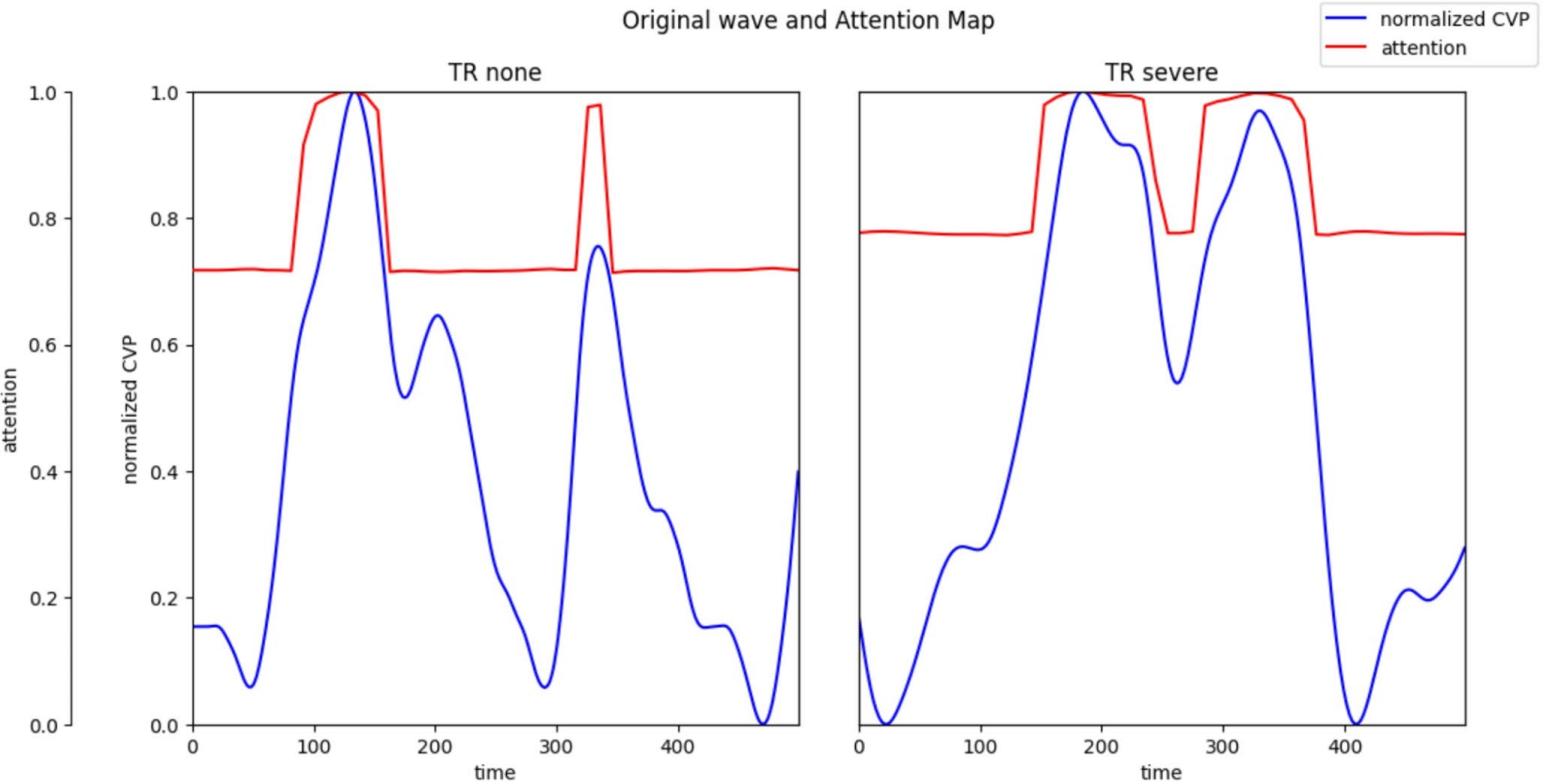
Transformer in Time Series model used to predict CVP waveform of the none and severe groups, and the prediction basis was visualized using attention Map. CVP, central venous pressure.

Transformer in Time Series was used to create a model to classify TR as none or severe. Figure 7 shows the confusion matrix of the models created using Transformer in Time Series. The accuracy was 0.97. The transformer in the time-series model was adapted for the waveforms with no and severe TR, and the basis for the decision was visualized on an attention map, as shown in Fig. 8. Attention was generally high; however, in severe TR, it was particularly high for the C and V waves. The attention was high for the V waves in the no TR group, and the area around the peak was considered to be an A wave. The accuracy and loss during model training are presented in Appendix 2.

## Discussion

This study identified a relationship between the severity of TR on echocardiography and CVP waveforms. First, $\:C\:wave\:-\:Y\:descent$ and $\:X\:descent-Y\:descent$ showed a significant trend in the Jonckheere–Terpstra test according to the severity of TR (*p* = 0.0019, 0.027 respectively). Notably, only $\:X\:descent-Y\:descent$ was found to have sufficient discriminative power in the none to moderate TR and severe TR groups, with an AUC of 0.83 (95% CI [0.68, 0.98]). The absolute pressure difference in $\:X\:descent-Y\:descent$ was considered statistically significant. Second, CVP waveforms for no TR and severe TR were identified using deep learning. The accuracy of the validation dataset was 0.97 in the Transformer in Time Series. Attention was particularly high for the C and V waves in the attention map for severe TR. In contrast, attention was high for V and A wave in the no TR group. The shapes of the C and V waves are considered important for deep learning.

The database of the recorded waveforms displayed on a biological monitor was analyzed to quantitatively assess the CVP waveform. This database includes all cases that underwent biometric monitoring in the operating room. Unlike previous case reports, as many as 436 cases were analyzed in the present study using this database.

The original CVP waveform contained artifacts, which were removed using k-Shape. These artifacts were removed as a preliminary step in the analysis. Although machine learning is suitable for dealing with large amounts of data at high speeds, it may not be able to remove artifacts perfectly. It is possible that waveforms without artifacts were mistakenly included in the group labeled as having artifacts, and waveforms with artifacts were mistakenly included in the group labeled as free of artifacts.

Differences were observed in the number of cases according to the severity of TR as the data were collected retrospectively. The group with the highest number of cases had 271 cases, whereas the group with the lowest number of cases had 11 cases. Individual differences in the groups with fewer cases may have influenced the results.

Simple indices were developed from the CVP waveform and analyzed statistically based on the severity of TR. Considering that the values of the indices increased with increasing severity of TR, the statistical test used was the Jonckheere–Terpstra test. In the Steel–Dwass multiple comparison test, $\:X\:descent-Y\:descent$ was found to differ significantly different between the none and severe groups, the trivial and severe groups, the mild and severe groups, and the moderate and severe groups. In contrast, $\:C\:descent-Y\:descent$ was found to differ significantly only between the none and moderate groups. This may be attributed to the limited number of severe cases included in this study, which may have prevented the development of a significant difference in the comparisons between the severe and other groups. Unlike large V wave ROC curves, $\:X\:descent-Y\:descent\:$was closely related to the severity of TR when absolute pressure differences were used.

Deep learning was used to handle the complex features of the CVP waveforms. Transformer in Time Series was used in this study as it can handle time-series data and visualize the basis for decisions. Since the training and validation data were categorized according to the case, the waveforms from the same case were not split into different groups. The CVP waveforms used for deep learning were normalized; therefore, absolute pressure data were lost; however, relative pressure comparisons and complex waveform shapes could be considered. As there were no similar models to serve as references, the choice of hyperparameters for deep learning was adjusted by observing the training results such that the accuracy was high, the loss function was low, and overfitting did not occur. The accuracy observed in the present study was 0.97, indicating that a good deep learning model for classifying TR as none or severe can be created. Owing to their high accuracy, the results of the attention map are also considered noteworthy. It has been shown that C and V waves may be important when considering relative pressure comparisons and complex waveform shapes. The attention map can display each waveform individually; however, it cannot display the results of all waveforms in the dataset together. Therefore, it is difficult to consider the results of the attention map for all waveforms.

X descent was considered important in the statistical analysis. However, C and V waves are considered important for deep learning. When considering only the absolute pressure difference, X descent was important; however, based on the results of deep learning, the C and V waves could also be important. The attention map shows the regions where the Transformer places weights in the calculation but not how it is calculated. It is difficult to identify the important features of a region with the use of deep learning. Whether the shapes of the C and V waves or their ratios were important could not be clarified further. Since TR regurgitation could be a holosystolic flow, it was not surprising that it affected the C wave, X descent, and V wave. The present study showed that there are changes in the C wave, X descent, and V wave depending on the severity of TR, as noted empirically.

This study had several limitations. First, as CVP is a low-pressure system, it is thought to generate more artifacts than high-pressure systems, such as arterial pressure and pulmonary arterial pressure. In addition, the coordinates of the CVP waveform vertices used during statistical analysis may have led to detection errors. Second, the severity of TR was classified according to preoperative echocardiography. Therefore, the severity of preoperative TR is not always equivalent to the severity of TR before surgery. In fact, there are reports that the severity of TR is higher in preoperative echocardiography compared to intraoperative echocardiography^5^. Thus, CVP waveforms should be compared with the echocardiography results measured in real time. However, it is said that the TR in preoperative echocardiography moderately correlates with the TR in intraoperative echocardiography^5^. While the severity of TR in our study may not be the same as the exact intraoperative severity, the relative relationship of severity is likely maintained. Therefore, we believe it is possible to discuss the characteristics of severe TR compared to mild TR.

In conclusion, this study demonstrated that the severity of TR significantly influences the shape of the CVP waveform by using statistical analysis and deep learning. This study demonstrated the potential of using deep learning to extract features from waveforms that can be managed by humans.

## Electronic supplementary material

Below is the link to the electronic supplementary material.

Supplementary Material 1

## Data Availability

The data analyzed during the current study are not publicly available due to privacy concerns of the individuals who participated in the study, but they are available from the corresponding author upon reasonable request.
