# Supplementary material for: Assessment of the relationship between central venous pressure waveform and the severity of tricuspid valve regurgitation using data science
Source: Sci Rep. 2024 Oct 22;14:24839. doi: 10.1038/s41598-024-74890-8 (PMC11496678; doi:10.1038/s41598-024-74890-8)
Supplement: Supplementary file 1 — Supplementary Material 1 [file 41598_2024_74890_MOESM1_ESM.docx]

**Appendix 1:** **Data Cleaning by k-Shape**

Artifacts from the original CVP waveform were removed using k-Shape [23]. Anomaly detection methods were applied for this purpose [24, 25]. A review of the literature did not reveal any method for removing artifacts from pressure waveforms; therefore, a machine-learning anomaly detection method used in engineering was applied [24].

k-shape is a clustering method for time-series data [23] that is based on a scalable iterative refinement procedure, similar to that used in k-means. Shape-based distance (SBD) distance was used as a measure of similarity in a k-Shape. Each waveform belongs to a cluster with the smallest SBD from the centroid of each cluster. The centroid of each cluster was the waveform with the minimum squared distance from the waveform belonging to that cluster.

The elbow method and the shape of the waveform of the centroid were used to determine the number of clusters as follows: The SBD of the waveforms from the centroid of each cluster was calculated, and the average was obtained. The averages of the total number of clusters from 1 to 15 were calculated and plotted. The number of clusters, for which increasing the number of clusters did not significantly reduce the mean SBD was determined. In addition, the number of clusters in which no new centroid waveforms appeared after increasing the number of clusters was used.

The waveforms were clustered according to the number of clusters. Normal and artifact clusters were then identified based on the shape of the centroid waveform. Waveforms belonging to the normal cluster within a specific SBD were considered normal [24, 25]. The specific SBD was determined to be 0.3. The threshold for the distance that narrows down the normal and artifacts within a normal cluster was determined by examining the CVP waveform, as there was no precedent to serve as a reference.

**Appendix 2: accuracy and loss of deep learning during training**

The accuracy and loss during model training in Transformer in Time Series are shown in Supplementary Figure S1 online.

**Supplementary Fig.S1**





The changes in accuracy and the change in loss function for each epoch for the training and validation data in Transformer in Time Series.
